# Research on Human Posture Estimation Algorithm Based on YOLO-Pose

**DOI:** 10.3390/s24103036

**Published:** 2024-05-10

**Authors:** Jing Ding, Shanwei Niu, Zhigang Nie, Wenyu Zhu

**Affiliations:** 1 Department of Physical Education, Gansu Agricultural University, Lanzhou 730070, China; dingj@gsau.edu.cn; 2 College of Information Science and Technology, Gansu Agricultural University, Lanzhou 730070, China; niusw@st.gsau.edu.cn; 3 Key Laboratory of Opto-Technology and Intelligent Control, Ministry of Education, Lanzhou Jiaotong University, Lanzhou 730070, China; 4 Intelligent Sensing and Control Laboratory, Shandong University of Petrochemical Technology, Dongying 257000, China; 2015017@sdipct.edu.cn

**Keywords:** deep learning, target detection, YOLO-Pose, human posture, drones

## Abstract

In response to the numerous challenges faced by traditional human pose recognition methods in practical applications, such as dense targets, severe edge occlusion, limited application scenarios, complex backgrounds, and poor recognition accuracy when targets are occluded, this paper proposes a YOLO-Pose algorithm for human pose estimation. The specific improvements are divided into four parts. Firstly, in the Backbone section of the YOLO-Pose model, lightweight GhostNet modules are introduced to reduce the model’s parameter count and computational requirements, making it suitable for deployment on unmanned aerial vehicles (UAVs). Secondly, the ACmix attention mechanism is integrated into the Neck section to improve detection speed during object judgment and localization. Furthermore, in the Head section, key points are optimized using coordinate attention mechanisms, significantly enhancing key point localization accuracy. Lastly, the paper improves the loss function and confidence function to enhance the model’s robustness. Experimental results demonstrate that the improved model achieves a 95.58% improvement in mAP50 and a 69.54% improvement in mAP50-95 compared to the original model, with a reduction of 14.6 M parameters. The model achieves a detection speed of 19.9 ms per image, optimized by 30% and 39.5% compared to the original model. Comparisons with other algorithms such as Faster R-CNN, SSD, YOLOv4, and YOLOv7 demonstrate varying degrees of performance improvement.

## 1. Background

The study of human posture is of great significance for understanding movement mechanisms, improving motor skills, and optimizing training programs. China has always been committed to improving the health of its people, and has emphasized adherence to the principle of prevention as the mainstay, optimization of the health service system, and enhancement of the non-medical health service model. The healthy growth of the body has a profound impact on the overall health of college students and is an important manifestation of the country’s comprehensive strength, as well as a key resource for the country’s sustainable development. Health education in colleges and universities, as an important component for the implementation of such a program, has the responsibility to promote correct health concepts and methods.

The main cause of poor body posture is an abnormal change in the alignment of the bones, which affects range of motion and places abnormal stress on muscles, joints, ligaments, and other tissues. In such unbalanced postures, the rest of the body “compensates” for the imbalance, but the balance is fragile and gradually leads to discomfort and pain. With increased awareness of the importance of managing body shape and health, the “pre-intervention” model of correction and rehabilitation is becoming more widely recognized, and effective methods such as exercise are being used to address the problem proactively, thereby reducing the risk of disease associated with poor posture.

Human posture estimation is an important computer vision task aimed at detecting human posture from images or videos, including the body’s joint positions and relative angles. This task is of great significance. Traditional 2D human pose estimation methods are mainly classified as bottom-up and top-down. Top-down methods first detect all human positions in the image and then estimate the pose information and size for each detected person independently. Bottom-up methods, on the other hand, first utilize a convolutional neural network (CNN) or its variants to extract features from the input image and then use a dense key point detector to detect all relevant key points and combine the human pose information based on the relationship between them. Human body posture estimation must judge and locate the target and interpret the target’s rotation angle in three-dimensional space. In traditional two-dimensional image recognition in the process of practical application, there are low light intensity, dense targets, serious edge occlusion, single application scenes, complex background of the target object, the target is occluded, recognition accuracy is poor, etc. All of these problems in human body posture estimation algorithms’ recognition speed and accuracy are challenging.

## 2. Introduction

### 2.1. Research Work by Relevant Scholars

With the advancement of deep learning and the increase in computational power, significant progress has been made in human pose estimation network models. Zheng et al. [1] (2021) proposed a purely Transformer-based method called PoseFormer for 3D human pose estimation in videos. This method comprehensively models the intra-frame human joint relationships and inter-frame temporal correlations to output accurate 3D human poses for the central frame. Liu et al. [2] (2021) introduced a novel multi-frame human pose estimation framework that leverages rich temporal cues between video frames to enhance key point detection. This method encodes the spatiotemporal context of key points through pose–time merging to generate an effective search range and computes bidirectional weighted pose residuals through a pose residual fusion module, effectively improving pose estimation. Li et al. [3] (2021) proposed an efficient and effective regression-based approach utilizing maximum likelihood estimation (MLE) for developing human pose estimation, modeling the output distribution using likelihood heatmaps. Zhang et al. [4] (2021) proposed a method that utilizes the YOLOv3 model to create a human pose estimation network, combining the squeeze-and-excitation network structure in High-resolution network (HRNet) residual architecture and improving the HRNet algorithm’s output of human key points. They designed a pose classification algorithm based on support vector machines (SVMs) to classify human poses in a classroom setting.

Li et al. [5] (2022) proposed a strided Transformer architecture to efficiently convert a long sequence of 2D joint positions into a single 3D pose. This method combines single-frame supervision and applies additional temporal smoothness constraints to generate smoother and more accurate 3D poses. Liu et al. [6] (2022) introduced an anisotropic Gaussian coordinate encoding method to describe the skeletal orientation cues between adjacent key points. This is the first time skeletal orientation cues have been incorporated into heat map encoding for human pose estimation (HPE) tasks. They also introduced multiple loss functions to constrain the output and prevent overfitting. They use Kullback–Leibler divergence to measure the difference between predicted labels and ground truth labels. This method demonstrates significant advantages over existing state-of-the-art models for human pose estimation, but it suffers from algorithmic complexity and poor robustness, making it difficult to apply in real-life scenarios. Yuan et al. [7] (2022) proposed a video-based fall detection and orientation estimation method based on human pose estimation. They predict the coordinates of key points for each person using a pose estimation network and then use an SVM classifier to detect falls. This approach can effectively be applied to fall detection and orientation estimation in videos. Lee et al. [8] (2022) proposed an OpenPose network and applied the DeepSort algorithm for multi-person tracking. This algorithm can identify the poses of each individual based on the single-frame joints obtained from OpenPose. However, the algorithm exhibits poor robustness and struggles to cope with the challenges of complex human pose estimation in current scenarios.

Su et al. [9] (2022) proposed a motion pose estimation algorithm based on OpenPose and trained it using the COCO dataset. Through comparison with standard poses, the study demonstrated the algorithm’s ability to accurately recognize various badminton action poses, with a recognition rate of up to 94%. Amadi et al. [10] (2023) introduced a novel and fully differentiable pose consistency loss method. This method is unaffected by camera direction and has shown improvements in single-view human pose estimators trained using limited labeled 3D pose data. Manesco et al. [11] (2023) proposed a novel approach called the domain unified method, aiming to address pose misalignment in cross-dataset scenarios through a combination of three modules on top of a pose estimator, including a pose transformer, uncertainty estimator, and domain classifier. Li et al. [12] (2023) presented a hybrid model that combines convolution and transformation models to address the inconsistency between the performances of key point localization with higher accuracy and overall performance.

### 2.2. Contribution of This Article

Building on the academic research of the aforementioned scholars and the practical engineering applications, this paper proposes a novel approach to address the challenges of severe occlusion at the edges, complex background, and low recognition accuracy caused by target occlusion in human pose estimation. The aim is to improve the detection speed, accuracy, and robustness of the model.

**Figure 1:**
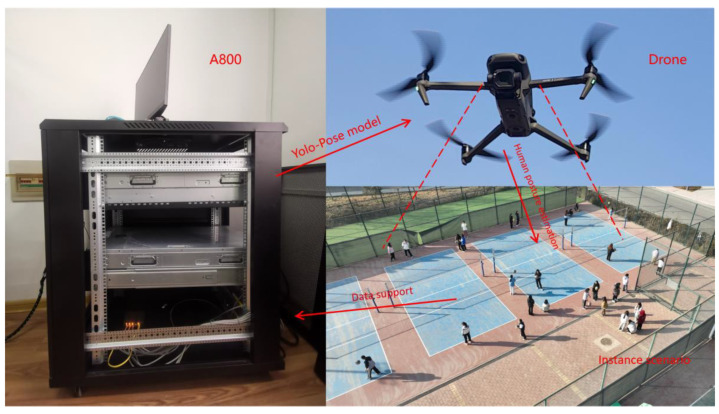
A summary of the research work in this paper.

Regarding the YOLOv5 model, which is currently a milestone in the industrial world, its lightweight and precision improvement give full play to its simplicity, ease of use, and high efficiency [13] and lay the foundation for its wide application in actual production, life, and industrial engineering. The summary diagram of this work is shown in Figure 1.

The main contributions of the research presented in this paper are as follows:

(1) The Backbone section introduces a lightweight GhostNet module to complete the generation of redundant features with a more economical linear transformation, thus greatly reducing the computational cost of convolution to lower the number of parameters of the model and reduce the arithmetic demand, making it more suitable for deployment on UAVs.
(2) The Neck part introduces the ACmix attention mechanism, which captures local features by convolution in the task of judgement and localization of the target by the model, so that it focuses on judging the human body’s bounding box convolution of local features to improve the detection speed.
(3) The key points in the Head part and the decoupling information of key points are optimized through the coordinate attention mechanism in order to solve the problems of complex target background and poor target occlusion detection accuracy and to improve the positioning accuracy of key points.
(4) The loss function and confidence function are improved to guarantee the robustness of the projection of the bounding box (BBox) for human pose estimation in complex scenes in order to improve the robustness of the model and prevent the occurrence of lagging, frame dropping, and video blurring problems [14].

## 3. Experimental Data

The resources for this experimental study consist of three components: the study object, the data collection and dataset, and the A800 deep learning GPU arithmetic server.

### 3.1. Research Object

A total of 227 undergraduate female students from Gansu Agricultural University, who were enrolled in the academic year of 2022, were randomly selected as the subjects of this study. Initially, a questionnaire survey was conducted to collect basic information regarding their height, weight, body fat percentage, daily routines, medical history, pain history, allergy history, lifestyle habits, and self-perception of body posture. It is worth noting that students diagnosed with definite spinal disorders, thoracic deformities, developmental abnormalities, as well as those exempt from physical education classes due to medical reasons, were not included in the scope of this research.

### 3.2. Data Collection and Dataset

The sample dataset collected in this study is derived from the daily learning and living scenarios of students at Gansu Agricultural University in Lanzhou, China. To ensure the diversity of images in the dataset, the experiment collected various complex scenarios including classroom learning, campus strolls, physical exercise, and laboratory activities. The methods of image collection in the dataset include the use of mobile devices and drones. The mobile devices utilize the Sony IMX866 large sensor camera, which significantly enhances color performance and image quality, with a resolution of 1279 × 1706 pixels, and a total of 600 images were collected. The unmanned aerial vehicle employs the Mavic 3 Cine model, equipped with a one-inch sensor and 20 million pixels, capable of capturing high-dynamic-range images, and possessing outstanding stability and endurance performance. The image resolution is 5280 × 2970 pixels, and 600 images were also collected. Both datasets, totaling 1200 images, were divided into training, testing, and validation sets in an 8:1:1 ratio.

**Figure 2:**
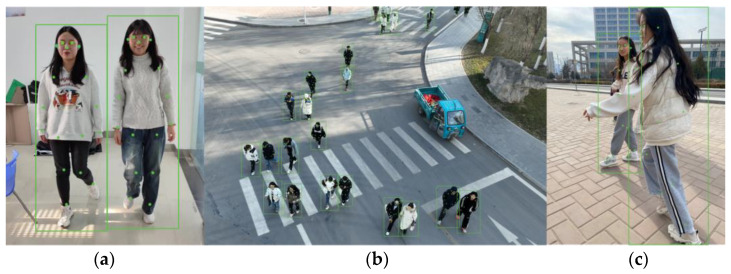
The schematic diagram of human pose key point annotation. (**a**) Laboratory; (**b**) Drone; (**c**) Physical Education Class.

In this study, we annotated human body pose key points using the Labelme software (version 1.8.6) and employed the COCO format for data annotation. The annotation was specifically performed on instances of the class “Person”. A total of 17 key points on the human body were annotated, including the nose, left eye, right eye, left ear, right ear, left shoulder, right shoulder, left elbow, right elbow, left wrist, right wrist, left hip, right hip, left knee, right knee, left ankle, and right ankle [15]. Each key point (C1, C2, C3) is stored in a JSON data format. Specifically, C1 and C2 represent 2D plane coordinate data of the human body pose estimation key points, while C3 serves as a decision identifier, indicating the presence or absence of the key point in the image. The annotated key points are illustrated in Figure 2.

### 3.3. Details of the A800 Deep Learning GPU Computing Power Server

The computational resource employed in this study was the A800 deep learning GPU server at the Intelligent Sensing and Control Laboratory of Shandong University of Petroleum and Chemical Technology in China. The server utilized for this purpose is the Wave Computing’s AI Server NF548M6. Its hardware configuration includes an Intel^®^ (Santa Clara, CA, USA) Xeon(R) Silver 4314 CPU @ 2.4 GHz × 64 as the CPU processor, equipped with 8 NVIDIA A100 GPUs. Graphical rendering utilized llvmpipe (LLVM 7.0, 256 bits), while the operating system was CentOS Linux 7 (3.28.2), with 128 GB of memory capacity and 2.048 TB of disk storage space. Python version 3.8 was employed, setting the learning rate for the neural network to 0.01, and using a batch size of 16 for image training. All computational experiments were conducted on this powerful computing platform.

## 4. YOLO-Pose Human Posture Estimation Algorithm

**Figure 3:**
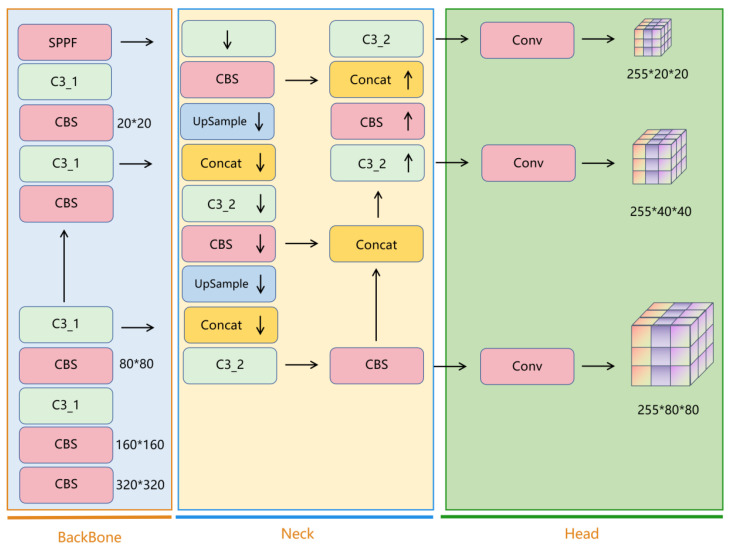
The YOLOv5 Network Model. Notes: * represents convolution.

The YOLO-Pose network structure, as illustrated in Figure 3, is built upon the foundation of the YOLOv5 network structure. The YOLOv5 network structure comprises three main components: Backbone, Neck, and Head [16], with the detailed flowchart depicted in Figure 3. The significance of YOLOv5 lies in its concise, user-friendly, and efficient characteristics, which have swiftly established its position in the industrial sector and enabled its widespread application in practical production fields. The scenario of this study involves conducting human pose detection tasks on unmanned aerial vehicles (UAVs), hence the utilization of the lightweight and low-computational-resource YOLOv5s.

**Figure 4:**
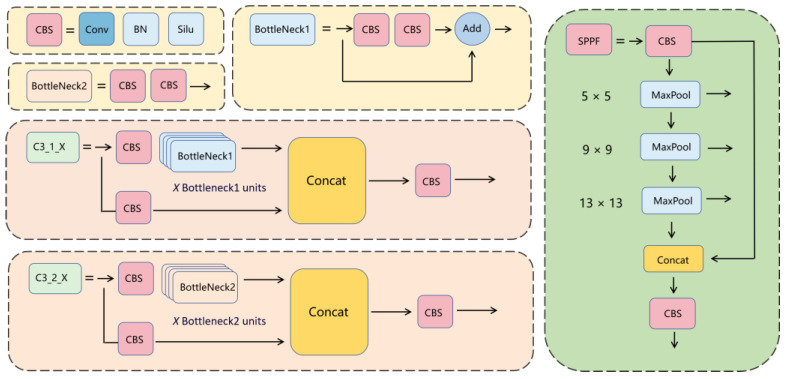
The Composition Structure of the Modules in the YOLOv5 Network Model.

In the Backbone, 4 C3_1s and 5 CBSs are utilized, with SPPF incorporated into the Backbone. The stride of all five CBSs is 2, resulting in a halving of both the height and width of the image after passing through the CBS. As shown in Figure 4, a CBS consists of a 2D convolutional layer, a BN layer, and a SiLU activation function. The distinction between BottleNeck2 and BottleNeck1 lies in the removal of the connection from input to output. Additionally, the difference between C3_1_X and C3_2_X lies in the use of BottleNeck1 in C3_1_X and BottleNeck2 in C3_2_X. C3_1 and C3_2 are collectively referred to as the C3 module, each employing three CBSs. Compared to CSPX, C3_X entails a smaller computational load. In YOLOv5, the authors transformed the BottleneckCSP module into the C3 module, which serves as the primary module for residual feature learning. It is composed of two structures: one acting on the Backbone main network and the other on the Neck module branch.

The spatial pyramid pooling (SPP) consists of SPP and SPPF, both of which are fundamentally similar modules that perform multi-scale transformations and fusion on feature maps. However, they differ slightly in structure. In SPPF, the input first passes through a CBS, followed by three layers of MaxPool, and eventually four output channels are merged to produce the final output through another CBS. It should be noted that, as opposed to SPP’s three pooling operations using window sizes of 5 × 5, 9 × 9, and 13 × 13, SPPF’s input for each pooling layer is derived from the previous layer’s output, with all three layers utilizing a 5 × 5 pooling window. Experimental results demonstrate that the computational load of the SPPF model is significantly smaller, leading to a substantial speed improvement.

**Figure 5:**
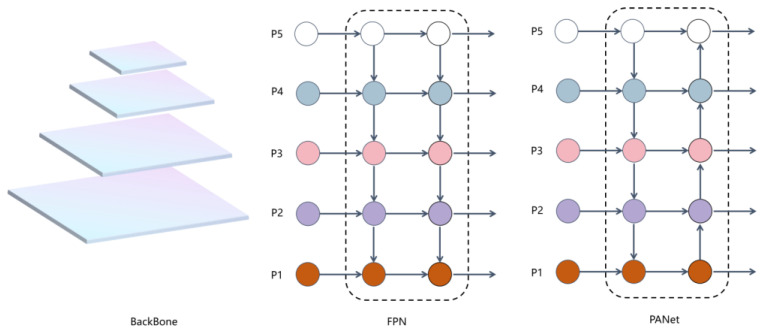
Schematic Diagram of Feature Fusion between FPN and PAnet.

The Neck section consists of 4 C3_1s, 4 CBSs, 4 Concts, and 2 UpSample modules. In the fusion pathways from top to bottom on the left side, there are two CBSs and two C3_2 modules; while in the fusion pathways from bottom to top on the right side, there are also two CBSs and one C3_2 module. This bidirectional feature fusion pathway is referred to as PAnet. As shown in Figure 5, PAnet merges high-dimensional features into low-dimensional features from top to bottom on the left side. Compared to the feature pyramid network (FPN), the bidirectional fusion structure of PAnet is more conducive to comprehensive feature integration. The Concat module is used for dimension concatenation, integrating feature maps with high dimensions lacking semantic information and low dimensions lacking detailed information. The UpSample module is used for upsampling, which effectively detects small objects and details. In the Head section, only one Conv module is retained, primarily to adapt the number of channels, uniformly transform dimensional information, and parse the channel information of the feature map into corresponding detection boxes and categories.

## 5. Improvement of YOLO-Pose Human Pose Estimation Algorithm

### 5.1. Backbone Section Introduces Lightweight GhostNet Module

**Figure 6:**
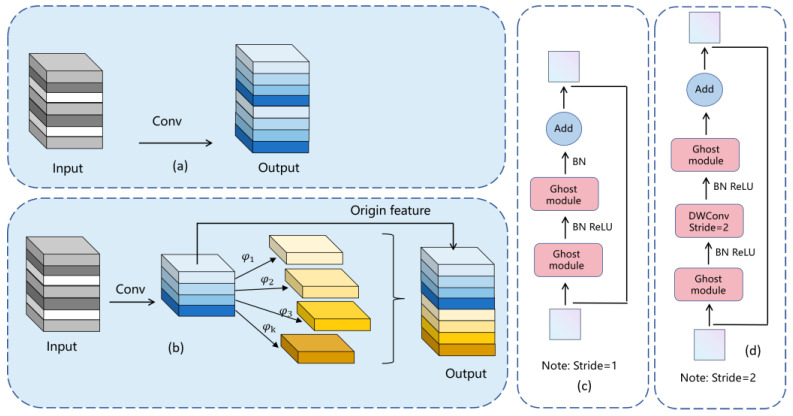
Lightweight Ghost Module Diagram. (**a**) Standard convolution. (**b**) Ghost Convolution. (**c**) Ghost module structure. (**d**) New Ghost module structure.

In practical application scenarios, standard convolution modules may generate a large number of approximate features, resulting in significant computational resource consumption. This becomes particularly problematic when deploying the model on unmanned aerial vehicles (UAVs) for human pose estimation tasks, as mobile devices on UAVs often have limited computational power, which can lead to stuttering and unsmooth model performance. To address this issue, we introduce the GhostNet module into the YOLO-Pose model. By utilizing more cost-effective linear transformations to generate redundant features, we greatly reduce the computational cost of convolution. Initially, we employ standard convolutions to generate m layers of original features, as illustrated in Figure 6a and computed using Equation (1).

(1) $$\gamma^{\prime }=X*f\;+b$$

In the formula, $\gamma^{\prime }\in \;R^{h\prime \times \omega \prime \times m}$ represents the output feature map, *b* represents the bias term [17], * signifies the convolution operation, and subsequently, $\gamma^{\prime }$ undergoes an inexpensive mapping. As shown in Formula (2), $y_{i}^{\prime }\in Y^{\prime }$ and $\varphi_{i,j}$ denote the j-th linear transformation of the source feature *i*. The schematic diagram is depicted in Figure 6b, where it is evident that $\varphi_{i,j}$ generates multiple corresponding Ghost features $y_{ij\;}$.

(2) $$y_{ij\;}=\varphi_{i,j}(y^{\prime }),\forall_{i},\ldots ,m,j=1,\ldots ,s$$

The standard convolution floating-point operation is denoted as n×h′× ω′×c×k×k, wherein *c* represents the number of input channels. In contrast, the Ghost convolution combines m(s−1)=n/s+(s−1) linear computations [18] with the standard convolution. The linear transformation convolves the kernel of size d×d. Hence, the computational ratio between the two can be expressed as Formula (3).

(3) $$R=\frac{nh\prime \omega \prime ckk}{(n/s)h\prime \omega \prime ckk+(s-1)(n/s)h\prime \omega \prime dd}=\frac{ckk}{s^{-1}ckk+((s-1)/s)dd}=\frac{sc}{s+c-1}\approx s$$

The Ghost convolution, compared to the standard convolution, increases the theoretical number of operations by a factor of *c* given d×d = k×k and s≪c. Leveraging the performance advantages of the Ghost module, two Ghost modules are combined to construct a new Ghost module structure, as illustrated in Figure 6c. The Backbone is formed by concatenating two Ghost modules in series. The role of the first module is to increase the feature dimension and expand the number of channels. The second Ghost module reduces the number of channels to match the number of input channels [19] and connects with the input through a shortcut to obtain the final output. Thus, the input and output dimensions of the new Ghost structure are the same, facilitating its integration into neural networks. When the stride is 2, a DWConv convolution layer with a stride of 2 is added between the two Ghost modules of the Backbone, which reduces the output feature map size to half of the input feature map size [20]. Two types of stride handling innovations provide greater flexibility for models to adapt to tasks of varying sizes and complexities. This study reconstructs the entire fusion network using the novel lightweight Ghost module to reduce model parameters and decrease computational requirements, making it more suitable for deployment on mobile devices, significantly enhancing the model’s usability and portability.

### 5.2. Neck Partially Introduces the ACmix Attention Mechanism

After introducing the lightweight Ghost module, the YOLO-Pose algorithm for human pose estimation has targeted detection and localization tasks. To further enhance performance, this study has also introduced the ACmix attention mechanism, which allows the network model to focus on feature information that is crucial for model performance, while ignoring irrelevant information and facilitating effective information exchange and propagation with other modules. The ACmix attention mechanism is a hybrid model that combines the advantages of self-attention and convolutional operations [21]. The core concept of this attention mechanism is to utilize 1 × 1 convolutions to perform most of the computations for both self-attention and convolutional operations, thereby enabling both global perception capability and the capture of local features through convolution.

**Figure 7:**
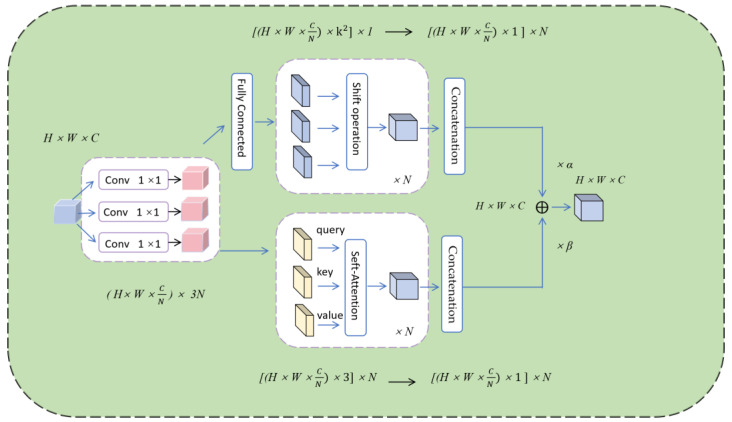
Structural diagram of lightweight ACmix attention mechanism.

According to Figure 7, the processing of the feature maps with a size of *H* × *W* × *C* is first performed through three 1 × 1 convolution projections, resulting in three sets of feature maps with sizes of *3* × *N*. Subsequently, convolution and self-attention operations are separately applied to these feature maps [22]. The convolution operation can be divided into two stages, namely Stage 1 represented by Equation (4) and Stage 2 represented by Equations (5) and (6).

(4) $$g_{ij}^{\sim (p,g)}=K_{p,q}f_{ij}$$

(5) $$g_{ij}=\sum_{p,q}g_{i,j}^{\left(p,q\right)}$$

(6) $$g_{i,j}^{\left(p,q\right)}=Shift(g_{ij}^{\sim (p,g)},p-\left\lfloor k/2\right\rfloor ,q-\left\lfloor k/2\right\rfloor )$$

In the above equations, $f_{ij}$ represents the feature vector of the input pixel, ⌊ ⌋ denotes the positional operation, *k* represents the kernel size, $g_{i,j}^{\left(p,q\right)}$ represents the feature map before projection, $g_{i,j}^{\left(p,q\right)}$ represents the feature map after projection, *Shift* represents the shift transformation, p,g represent the linear projection, $K_{p,q}$ represents the linear projection on each position, and $g_{ij}$ represents the total sum of the feature obtained after the aggregation operation.

The first stage involves projecting the input features onto different coordinate positions (p,q) according to the weight *K*. In the second stage, the projected mappings undergo horizontal and vertical shift operations separately based on $K_{p,q}$, and finally, all the mapped feature information is aggregated together [23].

Similarly, the self-attention operation can also be divided into 2 stages. The first stage is represented by Equation (7), and the second stage is represented by Equation (8).

(7) $$\left\{\begin{array}{c} q_{ij}^{\left(l\right)}=W_{k}^{\left(l\right)}f_{ij}\\ k_{ij}^{\left(l\right)}=W_{k}^{\left(l\right)}f_{ij}\\ v_{ij}^{\left(l\right)}=W_{k}^{\left(l\right)}f_{ij} \end{array}\right.$$

(8) $$g_{ij}=\begin{array}{c} N\\ ∥\\ I=1 \end{array}(\sum_{a,b\in N_{k}(i,j)}A(q_{i,j}^{\left(l\right)},k_{ab}^{\left(l\right)})v_{ab}^{l})$$

In the aforementioned equation, $W_{q}^{\left(l\right)}$ represents the input feature map matrix of the query at pixel (i, j), $W_{k}^{\left(l\right)}$ represents the input feature map matrix of the key at pixel (i, j), and $W_{v}^{\left(l\right)}$ represents the input feature map matrix of the value at pixel (i, j) [24]. $k_{i,j}^{\left(l\right)}$ is the feature mapping after key projection, $v_{i,j}^{\left(l\right)}$ is the feature mapping after value projection, $q_{i,j}^{\left(l\right)}$ is the feature mapping after query projection, and ∥ is the cascade of *N* attention head outputs. $N_{k}(i,j)$ denotes the region centered at pixel (i, j) with spatial width *k*, and $A(q_{i,j}^{\left(l\right)},k_{ab}^{\left(l\right)})$ denotes the corresponding weights in region $N_{k}(i,j)$. The feature mapping after the first stage of projection through three 1 × 1 convolutions is noted as query, key, and value, and finally the paths of the two operations are merged and summed to output as:

(9) $$F_{out}=\alpha F_{conv}+\beta F_{att}$$

In Formula (9), $F_{out}$ represents the final output of the path [25], $F_{conv}$ represents the output of the convolutional attention branch, and $F_{att}$ represents the output of the self-attention branch, which is used to measure the output weights. In order to achieve a balance between global and local feature information in the convolution and self-attention operations, this paper sets the models of α and β to 1, thereby enhancing the aggregation capability of intermediate mapping information for both modes and making the network more suitable for detecting small target information.

### 5.3. Optimizing the Head Section Key Points

**Figure 8:**
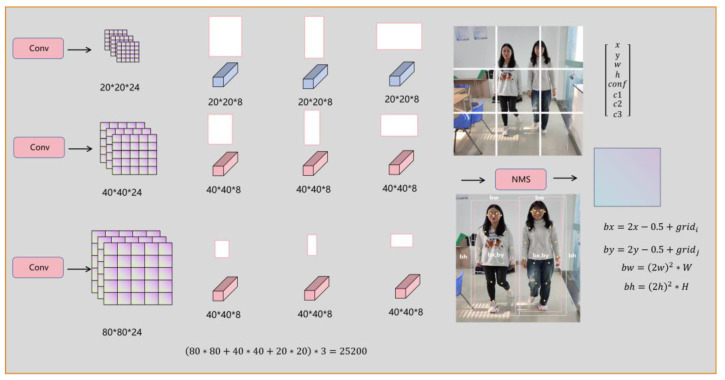
Schematic of YOLOv5 key point decoupling head. Notes: * represents convolution.

Key point detection is a task that is highly sensitive to position information, while human pose estimation is often affected by external factors such as lighting, resulting in missed and false detections of key points. In the original YOLOv5 network, the design of the key point decoupling head involves independent two-dimensional convolutional operations, as shown in Figure 8, predicting at three different scales (80 × 80, 40 × 40, 20 × 20). Each scale corresponds to three anchors, which in turn predict feature boxes at different scales of 80 × 80, 40 × 40, and 20 × 20. Therefore, the entire network predicts a total of 25,200 feature boxes. This design enables the network to more accurately identify and decode key point information. However, the large number of 25,200 feature boxes greatly wastes computational resources. For tasks involving human pose estimation by drones, computational resources are already scarce, thus non-maximum suppression (NMS) must be applied to filter out low-accuracy detection boxes, retaining only high-accuracy ones.

Each feature point in the sample images captured by drones has 8 feature channels, which include parameters representing the detection box, confidence (conf), 2D screen coordinates (C1 and C2) of the key points for human pose estimation, and an identification indicator for the existence of key points (C3). The detection box has 4 parameters, namely the center point (bx, by), width (bw), and height (bh), where the center point of the detection box falls within the grid at the center of the feature map [26]. During the computation process, the center point coordinates of the detection box are first calculated, with *gird_i_* representing the i-th column and *gird_j_* representing the j-th row. YOLOv3, YOLOv4, and YOLOv5 all employ anchor-based methods to compute the position of the detection box, although the formulas for calculating the center point coordinates (bx, by) as well as the width (bw) and height (bh) may differ slightly.

**Figure 9:**
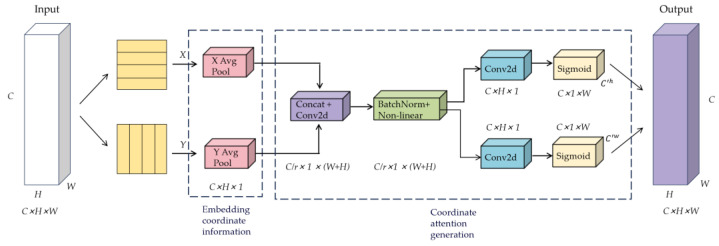
Schematic diagram of the structure of the key point attention mechanism.

This study further introduces the coordinate attention mechanism to optimize the decoupling of key point information and improve the accuracy of key point localization. The schematic diagram of the coordinate attention structure is shown in Figure 9, where *H*, *W*, and *C* represent the height, width, and channel number of the feature map [27], respectively.

The coordinate attention mechanism encodes the horizontal and vertical positional [28] information into channel attention, enabling the network to capture not only inter-channel information but also directional perception and position-sensitive information. Specifically, this mechanism consists of two steps: coordinate position embedding and coordinate attention generation. Firstly, we apply global average pooling with pooling kernels of size $\left[H,1\right]$ and $\left[1,W\right]$ to transform the feature map from a matrix of size [H,W,C] into a vector of size $\left[1,1,C\right]$. Following the global average pooling layer, we utilize 1D 1 × 1 convolutions to acquire inter-channel mutual information, with the size of the convolution kernels adjusted by an adaptive function. This adaptive function allows layers with more channels to engage in more inter-channel interactions. The specific adaptive function is described in Formula (10).

(10) $$k=\left|\begin{array}{ccc} \frac{{log}_{2}(c)}{\gamma } & + & \frac{\beta }{\gamma } \end{array}\right|$$

The channel adaptation performs optimally when γ = 2 and β = 1. We apply the adaptive function to the 1D 1 × 1 convolutions to obtain the weights for each channel in the feature pattern. Finally, by multiplying the normalized weights with the initial input feature pattern channels, we obtain the feature output $Z_{c}^{h}(h)$ and $Z_{c}^{h}(w)$ for the *c*-th channel at height *h* and width *w*.

(11) $$\left\{\begin{array}{c} Z_{c}^{h}(h)=\frac{1}{W}\sum_{0\leq i<W}x_{c}(h,j)\\ Z_{c}^{h}(w)=\frac{1}{H}\sum_{0\leq j<H}x_{c}(j,w) \end{array}\right.$$

In Equation (11), $x_{c}$ represents the input for channel *c*. The feature map obtained from Equation (11) is subjected to dimension concatenation and transformed into intermediate feature maps through operations like 1 × 1 convolution, batch normalization, and non-linear activation functions. This process yields intermediate feature mappings as shown in Equation (12).

(12) $$f=\delta (F_{1}([Z^{h},{Ζ}^{w}]))$$

In the equation, $f\epsilon R^{C∕r\times (H+W)}$ represents the intermediate feature containing both horizontal and vertical spatial information. φ represents the non-linear activation function. *Z^h^* and *Z^w^* represent the outputs of the concatenated feature map in terms of height and width, respectively. *r* denotes the reduction factor, *R* represents the set of real numbers, *C* represents the number of channels in the feature map, and *F*_1_ represents the convolution operation with a kernel size of 1.

Subsequently, the feature tensor *f* is split into two independent tensors, $f\epsilon R^{C∕r\times (r+H)}$ and $f\epsilon R^{C∕r\times (r+W)}$, along the height and width dimensions. Additionally, two 1 × 1 convolutions are employed to transform $f\epsilon R^{C∕r\times (r+H)}$ and $f\epsilon R^{C∕r\times (r+W)}$ into $F_{h}$ and $F_{w}$, respectively, ensuring that $f^{h}$ and $f^{w}$ have the same number of channels as the input feature tensor *X*. Afterward, the sigmoid activation function, σ, is separately applied to $g^{h}$ and $g^{w}$ to obtain attention weights along the height and width dimensions, as depicted in Equation (13):

(13) $$\left\{\begin{array}{c} g^{h}=\sigma (F_{h}(f^{h}))\\ g^{w}=\sigma (F_{w}(f^{w})) \end{array}\right.$$

Finally, the input feature map *X* is weighted by the attention weights $g^{h}$ and $g^{w}$ through a multiplication operation, resulting in the output of the coordinate attention module, denoted as $Y\in R^{C\times H\times W}$, as shown in the following equation.

(14) $$y_{c}(i,j)=x_{c}(i,j)\times g_{c}^{h}(i)\times g_{c}^{w}(j)$$

In Equation (14), $g_{c}^{h}$ and $g_{c}^{w}$ represent the attention weights of the feature map along the height and width dimensions, respectively, in the *c*-th channel. In this study, we have incorporated the coordinate attention mechanism into the conventional 2D convolution key point decoupling head. This mechanism enhances the sensitivity to the position of key points during the feature enhancement and prediction processes. It effectively addresses the challenges of accurate recognition and prediction in scenarios with complex backgrounds and occluded objects. Ultimately, it improves the accuracy of recognition and prediction.

### 5.4. Introduction of New Loss Function and Confidence Function

The YOLO-Pose network model outputs information including target class probabilities, coordinates of 17 key points, and confidence scores. In this study, the network training is conducted using the following loss function.

(15) $$L=\lambda_{pt}L_{pt}+\lambda_{conf}L_{conf}+\lambda_{id}L_{id}$$

In Equation (15), *L* represents the loss function, *L_pt_* is the coordinate loss, *L_conf_* is the confidence loss, and *L_id_* is the class loss. λ*_p_*_t_ is the weight for the coordinate loss function, λ*_conf_* is the weight for the confidence loss function, and λ*_id_* is the weight for the class loss function. The loss function, *L*, is composed of three components: the coordinate loss function, *L_pt_*, the confidence loss function, *L_conf_*, and the class loss function, *L_id_* [29]. The coordinate loss and confidence loss are computed using the mean square error function, while the class loss function is computed using the cross-entropy function.

In the early stages of training the network model, the precision of confidence prediction is low. At the beginning, it is necessary to set λ*_conf_* as 0 and gradually increase it for the units containing the target objects as training progresses. When dealing with units that do not contain the target object class, λ*_con_*_f_ is set to 0.1. The weight for the coordinate loss function, λ*_pt_*, is set as 1, and the weight for the class loss function, λ*_id_*, is also set as 1.

When training the network with the aforementioned loss functions, it has been demonstrated through empirical evidence that computing the IoU for calculating the loss function is extremely time-consuming. Therefore, this network proposes Equation (16) as a substitute for approximating the computation of IoU.

(16) $$c(x)=\left\{\begin{array}{c} \frac{e^{a}(1-\frac{D_{T}(x)}{d_{th}})-1}{e^{a}-1},D(x)<d_{th}\\ 0,otherwise \end{array}\right.$$

where $d_{th}$ represents the predicted distance of key points from the ground truth distance, D(x) represents the average error of key points in various bounding boxes (BBox) for human pose estimation, *a* denotes the hyperparameter scale factor for the current target, and *C(x)* represents the approximate IoU for the bounding box and predicted box. This approximation calculation greatly reduces the time consumption while mostly not sacrificing accuracy.

Building upon this foundation, the proposed model incorporates the calculation of variance for the error of each key point to ensure the robustness of the bounding box (BBox) projection for human pose estimation in complex scenes. The calculation formula is as follows:

(17) $$c\prime (x)=\frac{c\left(x\right)+\sum_{i=1}^{9}{\left(d_{i}-\bar{d}\right)}^{2}}{0,otherwise},D(x)<d_{th}$$

In our network model prediction, filtering is performed based on the confidence score and intersection-over-union (IoU) of the objects [30]. Similarly, in the three-dimensional space, it is necessary to analyze the confidence of the target objects. This network model employs the confidence function *f(x)* based on Euclidean distance to evaluate the deviation distance between the predicted pose of the target object and the ground truth pose. The updated formula is as follows:

(18) $$f(x)=\left\{\begin{array}{c} c(x)+\sum_{i=1}^{9}{\left(d_{i}-\bar{d}\right)}^{2}\\ 0,otherwise \end{array}\right.,D(x)<d_{th}$$

### 5.5. Improved YOLO-Pose Model

**Figure 10:**
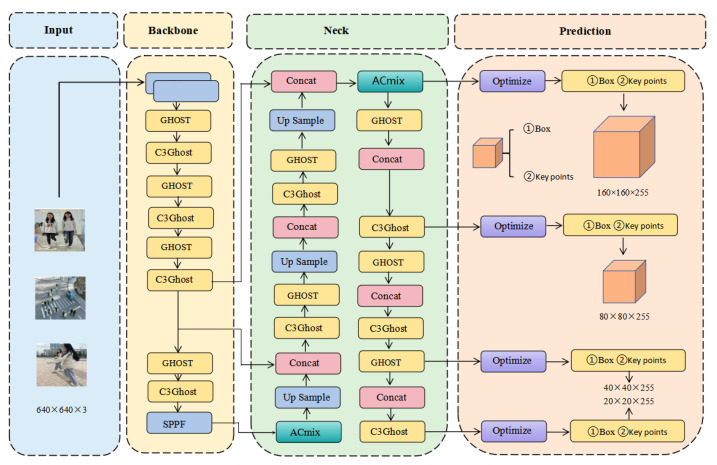
YOLO-Pose Network model diagram.

The improved YOLO-Pose model consists of four components: Input, Backbone, Neck, and Prediction [31], as shown in Figure 10. The Input component includes adaptive scaling, mosaic data augmentation, and anchor box calculation, where the adaptive image size is set as the default size of 640 × 640. YOLOv5 computes the optimal anchor box values for different training iterations. The mosaic data augmentation utilizes four images and combines them through random scaling, cropping, and arrangement. The purpose of anchor box calculation is to adjust the size and position of the correct targets in object detection. The Backbone component incorporates the GhostNet module, the Neck component introduces the ACmix attention mechanism to optimize the key point prediction in the Head component, and new loss functions and confidence functions are introduced. Through improvements in each module, the new YOLO-Pose model is formed.

## 6. Experimental Methods and Results

### 6.1. Network Training

This study adopts the stochastic gradient descent (SGD) as the core algorithm for network optimization. To maintain the stability of the deep layers in the model, a warm-up strategy is implemented during the training process. The initial learning rate is set to 0.0001 for predictive training, and it is decayed by a factor of 0.1 after every 150 epochs. The weight decay is set to 0.01. The transfer learning technique is employed, utilizing models trained on the ImageNet-1K and COCO datasets as pre-trained models. The gradient accumulation strategy is employed, with a batch size step of 4 and parameter updates performed every 16 steps. The training is conducted for 500 epochs, and loss and accuracy are sampled every 5 min per epoch.

### 6.2. Evaluation Indicators

In order to validate the speed, accuracy, and robustness of the improved YOLO-Pose model, the human pose estimation algorithm adopts the average precision based on the object key point similarity $L_{oks}$, as defined by the official MS COCO evaluation criteria. Specifically, $L_{oks}$ refers to:

(19) $$L_{oks}=\frac{\sum_{i}{exp}^{\left(-\frac{d_{i}^{2}}{2s^{2}k_{i}^{2}}\right)}\delta (v_{i}>0)}{\sum_{i}\delta (v_{i}>0)}$$

In Equation (19), i represents the annotated key point index, $d_{i}^{2}$ represents the squared Euclidean distance between the detected key point position and the ground truth key point position, $s^{2}$ represents the area occupied by the detected human body in the image, $k_{i}$ represents the decay constant used to control the key point category *i*. δ is the impulse function, indicating that the $L_{oks}$ value is only computed for visible relationship points in the ground truth annotations. $v_{i}$ represents the visibility of the i key point, where 0 signifies unannotated, 1 signifies annotated but occluded, and 2 signifies annotated and visible.

The evaluation of the algorithm’s object recognition accuracy for object category detection is conducted using precision (P), recall (R), and mean average precision (mAP) [32]. Precision (P) represents the proportion of correctly predicted samples among samples predicted as positive, as shown in Equation (20).

(20) $$P=\frac{TP}{TP+FP}\times 100\%\;$$

The recall (R) represents the proportion of correctly predicted samples among the actual positive samples, as shown in Equation (21).

(21) $$R=\frac{TP}{TP+FN}\times 100\%\;$$

The average precision (AP) is the area under the precision–recall curve, as indicated in Equation (12). The mean average precision (mAP) is defined as the average of AP values [33].

(22) $$mAP=\frac{\sum_{i=1}^{N}\int_{0}^{1}P(R)dr}{N}\times 100\%$$

**Figure 11:**
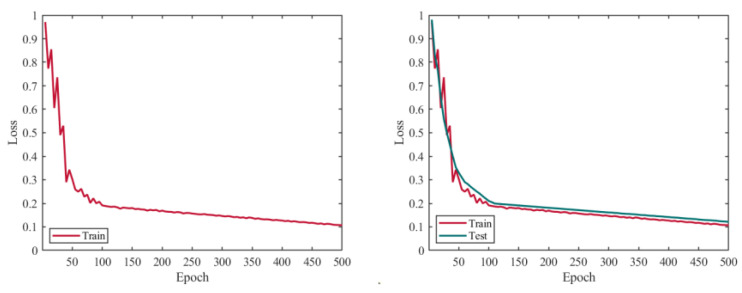
YOLO-Pose model training loss curve.

In the aforementioned equation, TP represents the number of samples correctly detected as the target class in the image, FP represents the number of falsely detected samples in the image, and FN represents the number of samples in the image where the target class was not correctly detected. The similarity losses of the target key points in the training and validation datasets, as well as the accuracy curves of various indicators in the training dataset, are shown in Figure 11. From the figure, we can observe that when the model is iterated 500 times, all the losses tend to stabilize and reach their minimum. At this point, all the accuracy metrics achieve their optimal values.

**Figure 12:**
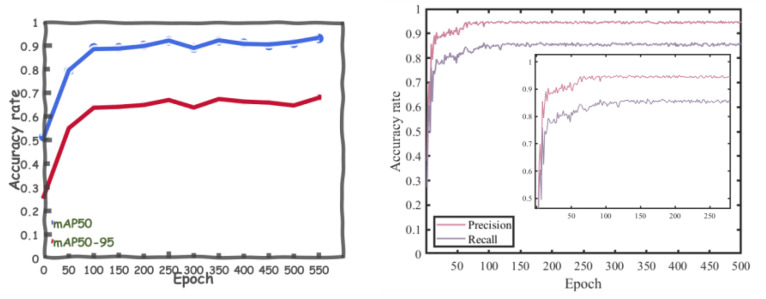
YOLO-Pose model accuracy loss curves.

In key point detection, we adopt mAP50 and mAP50-95 as evaluation metrics. Here, mAP50 represents the evaluation metric for single-object class detection accuracy when the threshold $L_{oks}$ is ≥0.5. mAP50-95 represents the average detection accuracy over 10 different thresholds, including 0.5, 0.55, …, 0.90, and 0.95, when using $L_{oks}$ as the threshold. Based on the results shown in Figure 12, the accuracy rates of both mAP50 and mAP50-95 steadily increase within the first 100 iterations, from 0.5 to 0.8 and from 0.2 to 0.5, respectively. After the 100th iteration, the model’s accuracy stabilizes, reaching around 0.8 for mAP50 and around 0.5 for mAP50-95. Precision and Recall stabilize around 0.94 and 0.85, respectively.

### 6.3. Ablation Experiment

In this study, the YOLOv5 network model was utilized, where improvements [34] were made by introducing the GhostNet module in the Backbone section and the ACmix attention mechanism in the Neck section, optimizing the key point prediction in the Head section, and incorporating new loss and confidence functions. To evaluate the impact of these improvements on the overall model performance, ablation experiments were designed. These experiments involved applying different modules to the original network to assess the effects of each component on the model’s performance enhancements.

Table 1 presents the improved YOLO-Pose algorithm for human pose estimation, which exhibits enhanced performance compared to the original model across various metrics. In terms of object detection, the improved model achieves an accuracy of 94.58% and a recall rate of 86.54%, representing improvements of 4.87% and 4.11% respectively, as compared to the original model. For key point detection, the improved model achieves mAP50 and mAP50-95 of 93.58% and 69.54% respectively, which demonstrate improvements of 5.24 and 5.05 percentage points over the original model. The improved model has a parameter size of 22.3 M. Furthermore, the detection time for a single image is 19.9 ms, showing respective optimization improvements of 30% and 39.5% compared to the original model, thereby meeting the requirements for real-time detection.

**Figure 13:**
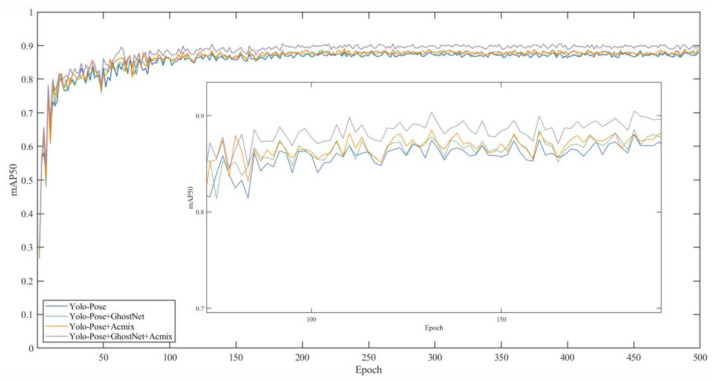
The training curve graphs for the ablation experiments of GhostNet and ACmix.

According to the graph, the training curves of Module1 GhostNet [35] and Module2 ACmix [36] are shown in Figure 13. It can be observed that the mAP50 performance of all four configurations shows a significant upward trend in the first 50 epochs. This indicates that the model quickly learns the patterns in the dataset during the early learning stages. Subsequently, the growth rate of the four curves begins to slow down and gradually enters a relatively stable state, indicating that the model starts to converge.

Throughout the entire training process, the configuration of YOLO-Pose + GhostNet + ACmix typically demonstrates the best performance (90.29%), followed by YOLO-Pose + ACmix (89.81%), YOLO-Pose + GhostNet (88.62%), and finally the baseline model YOLO-Pose (88.34%). This performance ranking suggests that combining GhostNet and ACmix effectively improves the model’s mAP50 performance.

### 6.4. Model Comparison

In order to further verify the effect of this paper’s algorithm after the improvement of light weight, speed, accuracy, and robustness, this paper’s algorithm is compared with other algorithms horizontally. As shown in Table 2, it is tabulated with classical target detection algorithms such as Faster R-CNN, SSD, YOLOv4, YOLOv7, etc., and the evaluated metrics are mAP50, mAP50-95, number of parameters, and detection time, respectively.

According to Table 2, the two-stage detection algorithm, Faster R-CNN [37], has the best detection accuracy and outperforms models such as SSD, YOLOv4, and YOLOv7. However, its detection speed lags far behind that of the one-stage detection algorithms. The SSD algorithm falls behind the YOLO algorithms, specifically YOLOv5 and YOLOv7, in both mAP50 and mAP50-95 metrics. YOLOv7 exhibits improvements of 0.92% and 1.54% in mAP50 and mAP50-95 metrics, respectively, compared to YOLOv4. Additionally, it reduces the detection time by 3.29 ms, but experiences an increase of 3.3 M in terms of parameters.

The YOLO-Pose algorithm proposed in this study achieves a higher detection accuracy than the Faster R-CNN algorithm by 2.09% in terms of the mAP50 metric. However, it lags behind by 0.67% in the mAP50-95 metric. This is because the two-stage detection algorithm introduces a CNN for feature extraction, enabling end-to-end training and utilizing bounding box regression to fine-tune the positions of human pose key points. Furthermore, our algorithm outperforms in terms of the Params metric by 5.3 M and in the detection time metric by 15.57 m/s. Overall, our algorithm strives to achieve an optimal balance among detection accuracy, parameter count, and detection time, taking into consideration the aforementioned characteristics.

### 6.5. Detection Effect

**Figure 14:**
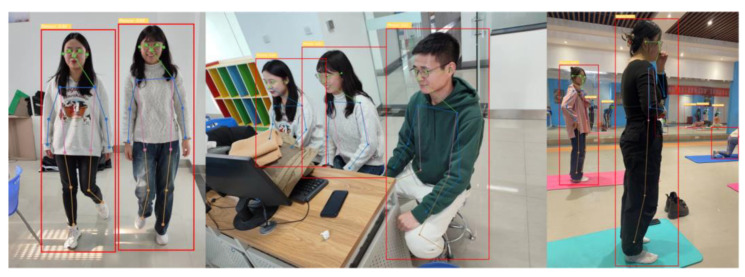
Visualization effect image 1 of YOLO-Pose model.

The visual results of the improved YOLO-Pose algorithm for human pose estimation proposed in this study are shown in Figure 14. We conducted detection for three different human poses, including standing, sitting, and participating in sports activities. It can be observed that the human key points are largely detected, even in scenarios involving small targets and complex sports poses. The algorithm demonstrates satisfactory performance in these challenging situations.

**Figure 15:**
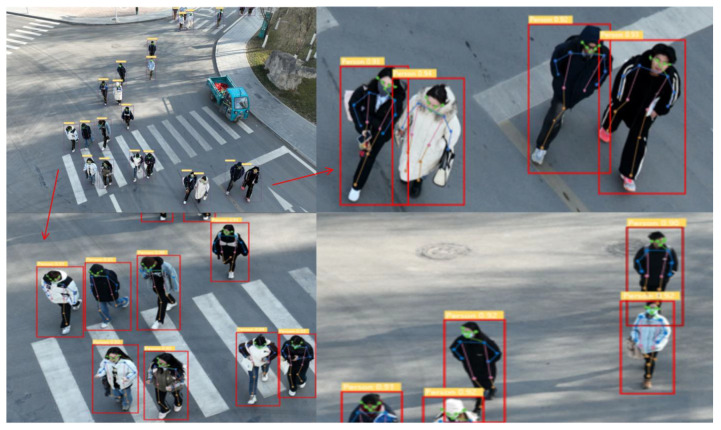
Visualization effect image 2 of YOLO-Pose model.

**Figure 16:**
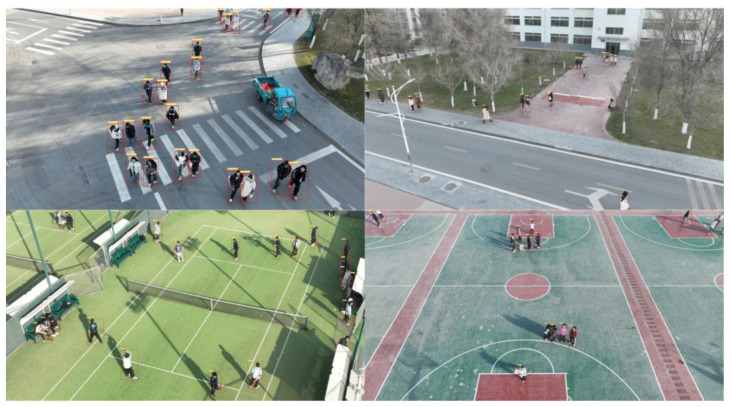
Visualization effect image 3 of YOLO-Pose model.

After deploying the YOLO-Pose model on an unmanned aerial vehicle (UAV), we conducted human pose detection in a different scenario. As shown in Figure 15, despite the challenges posed by the high number of targets and their small areas, the model was able to successfully accomplish the task of estimating human poses. Even when confronted with smaller target areas and more complex detection scenes, the model demonstrated its capability to largely complete the task, as depicted in Figure 16.

## 7. Conclusions

(1) Human pose estimation is a significant computer vision task; however, practical applications are often hindered by challenges such as low lighting conditions, dense target presence, severe edge occlusion, limited application scenarios, complex backgrounds, and poor recognition accuracy when targets are occluded. In this paper, we propose a YOLO-Pose model that leverages the lightweight and precision-enhanced features of the YOLOv5 object detection model, enabling its effective deployment on unmanned aerial vehicles (UAVs).
(2) Additionally, we employ transfer learning techniques by utilizing pre-trained models trained on the ImageNet-1K and COCO datasets to train our local dataset. In the YOLO-Pose model, we integrate lightweight GhostNet modules into the Backbone section to reduce the model’s parameter count and computational requirements, making it more suitable for deployment on unmanned aerial vehicles (UAVs) to accomplish specific human pose detection tasks. In the Neck section, we introduce the ACmix attention mechanism to enhance detection speed during object judgment and localization. Furthermore, we optimize the Head section’s key points by incorporating coordinate attention mechanisms to improve key point localization accuracy. We also enhance the loss function and confidence function to enhance the model’s robustness.
(3) The improved model demonstrates a reduction of 14.6 M parameters, an 8.47 ms decrease in detection time, a 5.24% improvement in mAP50, and a 5.05% improvement in mAP50-95. Notably, the parameter count and detection speed have been optimized by 30% and 39.5%, respectively, resulting in a detection speed of 19.9 ms per image. These enhancements enable the model to possess concise, user-friendly, and efficient features, making it suitable for monitoring students’ movement poses and assessing their body posture. The model provides valuable technical support by identifying and evaluating various types and levels of poor posture and offering low-cost and easily implementable intervention strategies for physical activities.

## Figures and Tables

**Table 1:** Network ablation test.

Module1	Module2	Module3	Module4	mAP50/%	mAP50-95/%	Params/M	Detection Time/ms
—	—	—	—	88.34	64.49	36.9	28.64
√	—	—	—	88.62	63.45	26.88	23.84
—	√	—	—	89.81	65.69	28.96	24.5
—	—	√	—	88.59	66.98	30.11	26.6
—	—	—	√	90.83	64.32	29.56	25.9
√	√	√	√	93.58	69.54	22.3	19.9

Notes: Module1 represents the introduction of the GhostNet module, Module2 represents the introduction of the ACmix module, Module3 represents the optimization of the Head part of the key point prediction, and Module4 represents the introduction of the new loss function and confidence function.

**Table 2:** Comparison of test results of different algorithms.

	mAP50/%	mAP50-95/%	Params/M	Detection Time/ms
Faster R-CNN	91.49	68.87	27.6	35.56
SSD	87.6	62.60	27.8	28.83
YOLOv4	90.64	64.94	25.9	28.64
YOLOv7	91.56	66.48	29.2	25.35
YOLO-Pose	93.58	69.54	22.3	19.99

## Data Availability

Data are contained within the article. You can send an email to the second author and corresponding author to request the data and code.
